# Clinical and Pathophysiological Insights Into Immunological Mediated Glomerular Diseases in Childhood

**DOI:** 10.3389/fped.2020.00205

**Published:** 2020-05-12

**Authors:** Antonio Mastrangelo, Jessica Serafinelli, Marisa Giani, Giovanni Montini

**Affiliations:** ^1^Pediatric Nephrology, Dialysis and Transplant Unit, Fondazione IRCCS Ca' Granda, Ospedale Maggiore Policlinico, Milan, Italy; ^2^Department of Clinical Sciences and Community Health, University of Milan, Milan, Italy

**Keywords:** review, primary glomerulonephritis, children, pathophysiology, histology, treatment, complement cascade

## Abstract

The kidney is often the target of immune system dysregulation in the context of primary or systemic disease. In particular, the glomerulus represents the anatomical entity most frequently involved, generally as the expression of inflammatory cell invasion or circulant or *in situ* immune-complex deposition. Glomerulonephritis is the most common clinical and pathological manifestation of this involvement. There are no universally accepted classifications for glomerulonephritis. However, recent advances in our understanding of the pathophysiological mechanisms suggest the assessment of immunological features, biomarkers, and genetic analysis. At the same time, more accurate and targeted therapies have been developed. Data on pediatric glomerulonephritis are scarce and often derived from adult studies. In this review, we update the current understanding of the etiologic events and genetic factors involved in the pathogenesis of pediatric immunologically mediated primitive forms of glomerulonephritis, together with the clinical spectrum and prognosis. Possible new therapeutic targets are also briefly discussed.

## Introduction

The glomerulus represents the anatomical entity of the kidney most frequently affected by autoimmune and inflammatory diseases. Glomerulonephritis (GN) is the major clinical and pathological expression of this involvement, which sometimes occurs in the context of systemic diseases, such as lupus nephritis and vasculitis. The tubular-interstitial compartment of the kidney is affected more rarely, for example in the presence of a clinical picture of interstitial nephritis.

Over recent decades, major advances have been made as regards our understanding of the pathogenesis of some pediatric forms of GN (Table 1). n Hypocomplementemic forms represent a wide spectrum of diseases ranging from acute (post-infectious GN) to severe forms of C3 glomerulopathies. Both the immunological and genetic mechanisms involved in their genesis have been identified, namely the autoantibodies against the complement cascade and mutations in genes encoding complement regulatory factors, which produce persistent activity of the alternative complement pathway and inflammatory responses. As regards normocomplementemic GN, the discovery of the roles of poorly O-galactosylated-IgA1 and phospholiphase A2 receptor can be considered as a real new insight. On the other hand, the new histological classifications have concretely contributed to a better understanding of patient prognosis and therefore to more tailored therapies. The most recent classifications of GN, which include histological, immunological, genetic and clinical aspects (biomarkers) are a direct consequence of the better understanding of the pathophysiological mechanisms underlying these pathologies (Table 1). This is also true for GN secondary to systemic diseases, such as Systemic Lupus Erytematosus (SLE), for which diagnostic criteria, histological classification, clinical and histological prognostic factors, specific biomarkers and therapeutic approaches have changed substantially over the last 10 years.

**Table 1:** Classification of primitive glomerulonephritis underlying the pathogenetic mechanism.

**Group of diseases**	**Glomerulonephritis**	**Pathogenesis**
Hypocomplementemic Glomerulonephritis	• Post-infectious GN (PIGN) • Immunocomplex-mediated membrano-proliferative GN • C3 glomerulopathies	• Immunocomplex deposition • Dysregulation of complement alternative pathway (acquired or genetic)
Normocomplementemic Glomerulonephritis	• GN due to IgA deposition (IgA Nephropathy, Henoch-Schönlein Purpura associated Nephropathy) • Glomerulonephritis due to *in situ* immune deposits (Membranous Nephropathy) • ANCA Associated Vasculitis Nephritis	• Abnormally glycosylated IgA deposition • Autoantibody-mediated • (*in situ*: anti-PLA2R, anti THSD7A) • Autoantibody-mediated (systemic: ANCA)
Rapidly Progressive Glomerulonephritis	• Immune complex related RPGN (PIGN, IgAN, IgAVN) • Antibodies anti-GBM deposition (Goodpasture Syndrome) • ANCA Associated Vasculitis Nephritis	• Immunocomplex deposition

In this review, we update the current understanding of the etiologic events and genetic factors involved in the pathogenesis of pediatric immunologically mediated primitive forms of GN, together with the clinical spectrum and prognosis (Table 1). Possible new therapeutic targets are also briefly discussed.

## Hypocomplementemic Glomerulonephritis

All GN types characterized by complement cascade activation are comprised in this group. Based on complement recovery time and clinical course, these forms can be classified as either **acute**: post-infectious GN (PIGN), or **chronic**: immune complex (IC)-mediated membrano-proliferative GN (IC-MPGN) and C3 glomerulopathies (C3G).

**Figure 1:**
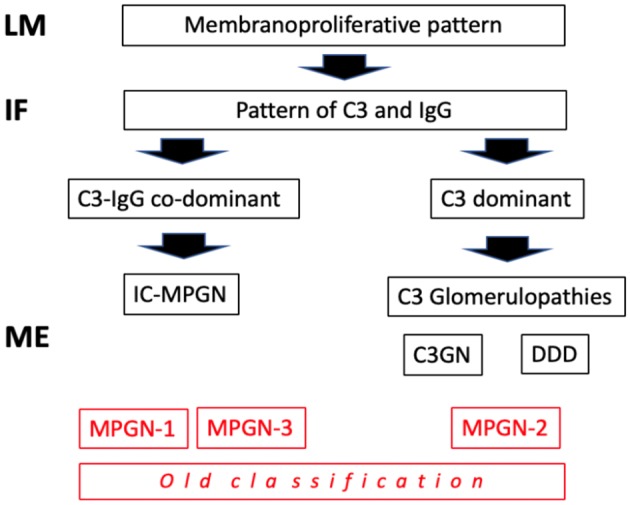
Classification of mempranoproliferative glomerulonephritis based on IF pattern. LM: light microscopy; IF, immunofluorescence; EM, electron microscopy.

Traditionally, the chronic forms were classified as type I, type II and type III membrano-proliferative GN (MPGN), according to the position of the deposits on electron microscopy (EM) (sub-endothelial, intramembranous, and sub-epithelial). Following a better understanding of the pathogenetic mechanisms involved (Table 1), there has been a reclassification. Types I and III MPGN, which exhibit deposits of IgG and C3 on immunofluorescence (IF), are now considered as MPGN caused by IC (IC-MPGN), while type II MPGN, also known as dense deposit disease (DDD), and all the forms with isolated/predominant C3 IF-deposits, are considered as C3G (Figure 1). Unlike MPGN, which is characterized by classical complement pathway (CCP) activation by IC deposition, C3G are associated with acquired or innate dysregulation of the alternative complement pathway (ACP).

### Post-infectious Glomerulonephritis

Post-infectious GN, which is triggered by a preceding infection, is frequently seen in children. It is most often caused by group A ß-hemolytic streptococci, while several other bacteria and viruses can also act as a trigger (1). In its classic form with gross hematuria, it affects 0.5–2 children/100,000 annually, although the pauci-symptomatic form, with microscopic hematuria, is up to 19 times more frequent and may remain undiagnosed (2, 3). Its incidence has drastically decreased in industrialized countries due to antibiotic use and improved sanitation, however it is still very common in developing countries, where the skin is the most prevalent site of infection (1).

#### Clinical and Laboratory Features

Typically, the disease affects children aged between 5 and 12 years; it is very rarely seen in children younger than 2 years because of the lower incidence of ß-hemolytic streptococcal infection in this age group and a reduced ability to produce IC.

The typical clinical presentation of PIGN is a nephritic syndrome with hematuria and proteinuria associated with signs of water retention (edema, hypertension). An increase in urea and creatinine values is often present, while a decrease in the C3 fractional complement values is the rule. Neurological and cerebral symptoms are frequently observed (10–30%) (4).

#### Natural History and Prognosis

In almost all cases, PIGN resolves spontaneously. Patients with typical post-streptococcal GN following a pharyngitis infection usually have a short illness, with rapid resolution (up to 7–10 days). Proteinuria disappears within 3 months in almost all cases, while microscopic hematuria may persist for up to 2 years (4). The persistence of hypocomplementemia beyond 8–12 weeks indicates a chronic form of GN (5) and prompts the need for further diagnostic testing, such as renal biopsy, the indications for which are shown in Table 2.

**Table 2:** Indications for renal biopsy in case of nephritic syndrome.

1.	Persistence of oligo-anuria beyond 7 days from onset
2.	Persistence of renal failure beyond 10 days from onset or rapidly progressive renal failure
3.	Persistence of nephrotic syndrome beyond 2–3 weeks from onset
4.	Persistence of hypocomplementemia over 12 weeks from onset
5.	Recurrence of gross hematuria after more than 1 month from onset

*Pathogenesis* Seven to 10 days post infection, circulating immune complexes (CICs) are formed and deposited in the glomeruli, causing leukocyte recruitment and ACP activation. Typically, once the infection disappears, the production of antibodies and new CICs decreases and the deposited ICs are cleared. As a consequence, clinical signs resolve within days to weeks (5). The risk of developing PIGN after a streptococcal infection has been calculated to be as high as 15% during epidemics. The trigger antigen has not yet been identified. The two enzymes that have been studied the most are glyceraldehyde 3-phosphate dehydrogenase, a plasmin receptor protein, and streptococcal exotoxin B (1).

Some studies have demonstrated that there are similarities between early C3G and PIGN. Streptococcal infection may trigger C3G (6–11). Several reports have demonstrated the presence of the same plasmin receptor protein in other complement mediated glomerulopathies (12, 13). The histological appearance of C3G may change with time. Early renal lesions in C3G could be difficult to distinguish from those seen in PIGN. Furthermore, the progression of glomerular involvement in a second biopsy from patients with persistent PIGN suggests a possible transformation into C3 glomuerulonephritis (C3GN). Finally, it is important to consider that the presence of inherited defects in the ACP may influence the prognosis of a patient with PIGN as it can cause delayed recovery, with development of segmental glomerular sclerosis and progressive chronic kidney damage (14).

#### Renal Histopathology (Figure 2)

Due to its specific pathogenesis, the most frequent histological picture of PIGN on light microscopy (LM) is that of endothelium-mesangial proliferative GN, with the presence of immunoglobulin-G (IgG) and C3 deposits [starry sky distribution that reflects the irregular coarse distribution along the glomerular basement membrane (GBM)] on IF. The picture can be complicated by epithelial cell proliferation (crescents) or an evident pattern of extra capillary proliferative GN (crescentic GN), which worsens the prognosis. Electron microscopy typically shows subepithelial deposits (humps).

**Figure 2:**
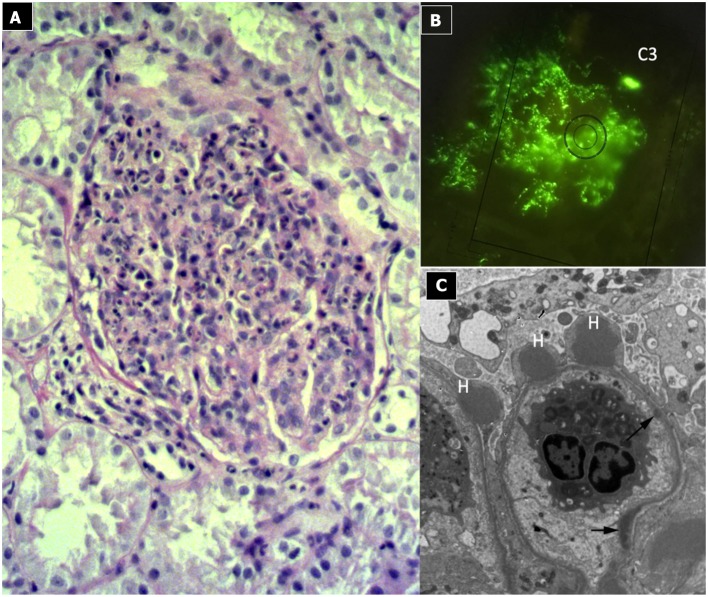
Post-infectious glomerulonephritis. **(A)** LM (PAS stain) shows endothelium-mesangial proliferative GN, with global increase of mesangial cellularity and matrix deposition. **(B)** IF: Starry sky distribution of C3. **(C)** EM: typical subepithelial deposits (humps).

#### Treatment

Post infectious GN can be prevented by the early onset of antibiotic therapy, in which case the respiratory or skin infections may not be so clinically evident. For this reason, in the context of epidemic infection, patients with clinical evidence of PIGN have to be treated as though they have an active infection (15).

Treatment with steroids or cytotoxic agents might have a role in severe or rapidly progressive diseases (Table 3). However, no randomized trials have been conducted on the subject (1).

**Table 3:** Therapeutical options for glomerulonephritis.

**Diseases**	**First line treatment**	**Other drugs**
PIGN	Steroids or cytotoxic agents (1) *(severe/rapidly progressive)*	Antibiotics (15)
MPGN	Treatment for underlying disease (16)*(secondary forms)* RAAS blockers (17) *(mild idiopathic form with low proteinuria)* Oral prednisone (16, 18, 19) *(idiopathic form with nephrotic proteinuria or kidney function deterioration)* pulse intravenous methylprednisolone + oral prednisone + cyclophosphamide (19) *(crescentic forms)*	RAAS blockers + cyclosporine *(steroid-resistant forms)* MMF (19) Rituximab (19)
*C3G*	“wait-and-see” (20) *(mild disease without nephrotic proteinuria)* Oral prednisone + MMF (21–23) *(moderate disease)* Pulse intravenous methylprednisolone (21) *(severe disease)* Eculizumab (24, 25), plasma exchange (26–28) fresh plasma infusion (27, 29, 30) *(RPGN or severe disease)*	calcineurin inhibitors (31, 32) rituximab (23, 33, 34) *(C3Nef antibodies)* C3 inhibitors, C5a inhibitors and C5aR antagonists (35–39) RAAS blockers (40)
*IgAN/IgAV*	RAAS blockers (41, 42) *(mild disease)* RAAS blockers + corticosteroids (Pozzi's protocol) *(moderate disease)* (42–46)	Cyclophosphamide (42, 47, 48) *(RPGN forms)* Eculizumab (48, 49) Tonsillectomy (50, 51), *(IgAN)* Azathioprine,MMF, cyclosporine (52) *(steroid-resistance forms)*
*MN*	“wait and see” policy (53) *(forms without kidney failure)* RAAS blocker (54–56) (persistent proteinuria)	Cyclophosphamide/ chlorambucil/cyclosporine (57) *(steroid-resistance forms)* Rituximab/ofatumumab (58, 59)
*AAV*	Pulse intravenous high dose steroids + cyclophosphamide (60, 61)	Rituximab (60, 61) *(refractory or relapsing form)* Plasma exchange *(renal failure or alveolar haemorrhage)* Low dose steroids + azathioprine, rituximab or MMF *(maintenance phase)*

### Immune Complex-Mediated Membrano-Proliferative Glomerulonephritis

This condition is also known as mesangio-capillary or lobular GN.

Membrano**-**proliferative GN can be primary (idiopathic) or secondary to different diseases, (62, 63) such as autoimmune disorders, monoclonal gammopathies, and infections, the most frequent being hepatitis C in adults (64). The idiopathic form is rare. While in adults its incidence has decreased over time (65), in children it has remained constant, likely due to the greater number of triggering infections (66).

#### Clinical and Laboratory Features

In children, IC-MPGN has a variable clinical presentation. It may include microscopic or gross hematuria, and varying degrees of proteinuria, from absent to nephrotic range (67). Impairment of renal function is frequent. Typically, complement activation tests show a reduction in levels of serum C3, C4, AP50, and CH50. Anemia and arterial hypertension can also be present at disease onset. In secondary forms of IC-MPGN, the symptoms of the underlying disease can overlap the clinical presentation.

#### Natural History and Prognosis

The course of the disease differs substantially. The important prognostic factors, reported in both adults and children, include deterioration of renal function, overt nephrotic syndrome (NS), severe glomerular hematuria (>50 red blood cells per high power field) and the presence of crescents or chronic lesions on kidney histology. Conversely, in secondary forms, identification of the underlying disease represents a favorable prognostic factor as it leads to the possibility of specific treatments (16). In pediatric patients, large population studies are lacking. The Mexican National Institute of Pediatrics experience reported that treatment with methylprednisolone slowed the progression of kidney damage, while persistent anemia and macroscopic hematuria favored kidney failure. Furthermore, the lower the albumin and/or glomerular filtration rate (GFR) levels, the higher the probability of developing chronic kidney disease (CKD) (63).

The Japanese focused on a prevention program: Yanagihara et al. reported a renal survival rate of 100% at 10 years, using high-dose steroids, with no severe adverse effects. Furthermore, they reported that detection through school screening and the early initiation of therapy improved prognosis (68). Finally, it has been reported that around 40% of children and young adults presenting with renal function impairment may progress to end-stage renal disease within 10 years (67).

#### Pathogenesis

Immune complex-mediated MPGN is typically caused by a chronic antigen stimulus with persistence of CICs (11, 16, 69, 70). The most frequent underlying disorders include chronic infections (hepatitis B/C, streptococcal and mycoplasma infections), autoimmune diseases (SLE, scleroderma, Sjögren syndrome) (60, 71–79), while monoclonal gammopathies are exceptional in children (80, 81).

Regardless of the trigger, CICs activate the CCP, leading to the production of chemotactic factors (C5a), opsonins (C3b) and the membrane attack complex (MAC, C5b-9), causing damage to mesangial and endothelial cells, with consequent chemotaxis of inflammatory cells. In response to damage, an intense cellular proliferation occurs, with basal membrane neoformation that traps the ICs.

#### Renal Histopathology (Figure 3)

Light microscopy shows a diffuse and global increase in glomerular tuft cellularity (lobular pattern), a marked and diffuse thickening of the glomerular capillary walls that often reveals a duplicity of the GBM on staining (double contour or tram track feature), and a diffuse and global reduction in the capillary lumens. Focal crescents may also be noted in around 10% of patients. Morphologic changes in the tubules and interstitium may also occur. Immunofluorescence shows co-dominance of both IgG and C3 and occasionally IgM (e.g., hepatitis C related MPGN) or a “full house” pattern (including IgG, IgM, IgA, C1q, C3, C4 and kappa and lambda light chains) as seen in SLE.

**Figure 3:**
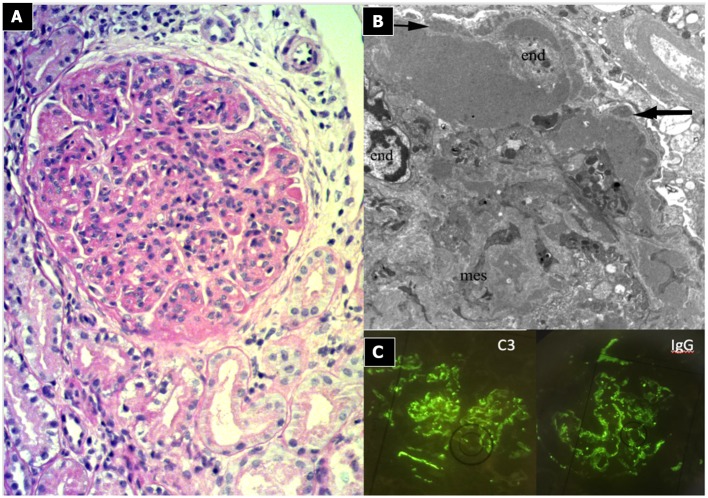
Immunocomplex-mediated membrano-proliferative GN. **(A)** LM (PAS stain): diffuse and global increase of glomerular tuft cellularity (lobular pattern) with thickening of the glomerular capillary walls (membranoproliferative pattern). **(B)** Arrows indicate GBM splitting due to mesangial interposition (white arrow where) and presence of mesangial and sub-endothelial deposits (black arrow). **(C)** IF shows IgG and C3 co-dominant positivity.

#### Treatment

Patients with IC-MPGN secondary to chronic infection or other autoimmune/myeloproliferative disorders should be treated for the underlying disease (Table 3) (16). There is no available adult or pediatric randomized trial data upon which to base treatment for patients with idiopathic IC-MPGN. It is important to consider renal function, severity of proteinuria and the presence of crescentic GN in the decision-making algorithm. The Kidney Disease Improving Global Outcomes (KDIGO) guidelines recommend, on limited evidence (17), inhibition of the renin-angiotensin-aldosterone system (RAAS) in mild cases of the disease, with low proteinuria (<500 mg/day) and normal renal function, in both adults and children. In cases of nephrotic proteinuria with normal renal function, a course of prednisone (1 mg/kg/day for 12–16 weeks) is suggested (16), even if in the literature there are no confirmatory RCTs (18). RAAS blockers and cyclosporine should be considered in instances of steroid-resistance. Steroids may also be useful when deterioration of renal function without crescents on biopsy is observed, while crescentic GN should be treated with pulse intravenous methylprednisolone followed by oral prednisone and cyclophosphamide. It is important to highlight the fact that these recommendations are based on weak evidence. Recently introduced drugs, such as mycophenolate mofetil (MMF) and rituximab, do not appear to improve survival compared to cyclophosphamide (19).

In our experience, long-term high dose alternate-day oral prednisone (1 mg/kg/alternate days) after steroid pulse induction therapy (methylprednisolone 15 mg/kg/dose for 3 days) is a useful therapy. It is rarely necessary to add an immunosuppressive agent (e.g., MMF) as a first-line drug, though this may be due to the lack of experience in the literature regarding the use of this drug.

### C3 Glomerulopathies

C3G are the pathological entities characterized by isolated C3 deposits, with minimal or no immunoglobulin deposition. The first international C3G meeting took place in Cambridge (UK) in 2012. The following year, the first consensus report on the definition, management, and treatment of C3G was published. The best-known forms are DDD and C3GN, based on EM findings and disease severity (61).

As this entity has been described relatively recently, its exact prevalence is difficult to estimate. The incidence of C3GN has been reported as 1–2 cases per million inhabitants (82), with a greater prevalence in the younger age group and a slight male preponderance (83, 84).

#### Clinical and Laboratory Features

The clinical spectrum is heterogeneous, ranging from mild disease with asymptomatic microscopic hematuria with or without proteinuria, to severe disease with a nephritic/nephrotic picture. Hematuria has to be considered pathognomonic of the disease. The presentation at onset is similar to DDD and C3GN (20), with minor differences reported in some series (84–86). Evidence of a preceding infection is present in 28–54% of patients, (11, 84, 87) many of whom were initially incorrectly labeled as PIGN. However, PIGN has more severe renal impairment and lower serum C3 levels at disease onset (88). In C3G, extra-renal manifestations such as acquired partial lipodystrophy, ocular deposits of C3 (Drusen) and thrombotic microangiopathy may occur (83, 85, 89).

#### Natural History and Prognosis

Most C3G patients have a chronic course, with persistent ACP dysfunction (low C3 levels in up to 75% of cases) with normal or slightly reduced C4 concentrations (11). Published data on the clinical course are, to date, few and conflicting. This is because the real prognosis varies according to the characteristics of the reported cases and the length of the follow up considered. However, clinical expression is so variable that it can independently affect prognosis (11, 83, 84). Nonetheless, the following risk factors have been identified: creatinine values >1.5 mg/dl at onset, renal histology showing crescents, severe arteriolar sclerosis, or DDD. The recurrence rate of the disease following transplantation was 50–55% for DDD and 43–67% for C3GN, (83, 84, 90, 91) with secondary graft loss in 50% and graft survival rates of 94–69–28% at 1, 5, and 10-years, respectively (91).

#### Pathogenesis

**Figure 4:**
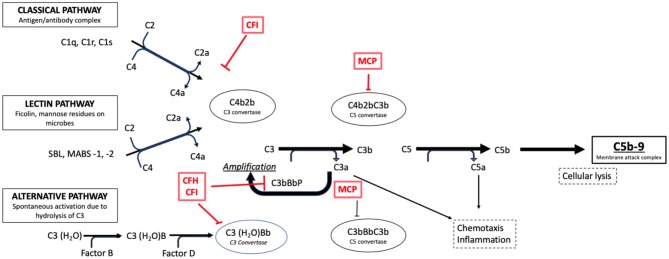
Activation pathways of the complement system in normal subjects. It is possible to note the complement regulation factors, such as CFH and CFI, and their point of action along the cascade phases (e.g., the C3 conversion for the CFH and CFI factors).

Both DDD and C3GN are driven by the dysregulation (acquired or congenital) of the ACP, however the specific mechanisms are unknown (Figure 4). It has been suggested that dysregulation of earlier components at C3 levels is implicated in DDD, whereas the late/terminal components are implicated in C3GN (84, 92). This could explain the aggressive course seen in DDD (10).

Regardless of the mechanisms, the outcome is hyperactivity of C3 convertase which causes a persistent C3 cleavage into C3a and C3b, maintaining ACP activity. The excessive formation of C5b-9 or MAC determines the cytotoxic effects responsible for cellular lesions. Acquired C3G is considered an autoimmune disease, given the presence of autoantibodies that block the function of complement cascade proteins. In 1965, Spitzer et al. were the first to describe the autoantibody directed against the C3 convertase of the ACP, C3 Nephritic Factor (C3NeF) (93), which was subsequently reported in up to 86% of patients with DDD and 46% of those with C3GN (83).

However, the exact role of C3NeF is incompletely understood: levels may vary regardless of disease activity, and be transiently present in PIGN (87, 94). In recent decades, some authors have described antibodies against Complement Factor H (CFH), the main regulatory protein of the ACP and, more rarely, against Factor B (CFB) and C3b (95). More recent data suggest a role for C4NeF, an autoantibody recognizing C4b2a, the C3 convertase for the CCP, in driving complement dysregulation in patients without other risk factors (96). The regulatory proteins of the ACP are genetically mutated in the congenital forms. In such cases, C3G may develop in the presence of a trigger. Mutations in the CFH gene, first described in 2006 in two brothers (29), are the most frequent. Other mutations in genes encoding C3, complement Factor I (CFI), CFB, factors related to CFH, CFHR1–5 and CD46 (MCP) have been associated with C3G, as already described in atypical hemolytic-uremic syndrome (97, 98). In particular, mutations in CFHR5 define an autosomal dominant disease termed CFHR5 Nephropathy, endemic among people of Cypriot ancestry and characterized by synpharingitic hematuria with a poor prognosis in male patients (99, 100). Mutations of complement factors have been described in up to 17% of patients with C3GN and 20% of patients with DDD (83).

Currently, no genotype-phenotype correlation has been recognized (20). It is strongly recommended that genetic testing be performed and interpreted by a center with expertise in atypical hemolytic-uremic syndrome and C3G (21).

#### Renal Histopathology

**Figure 5:**
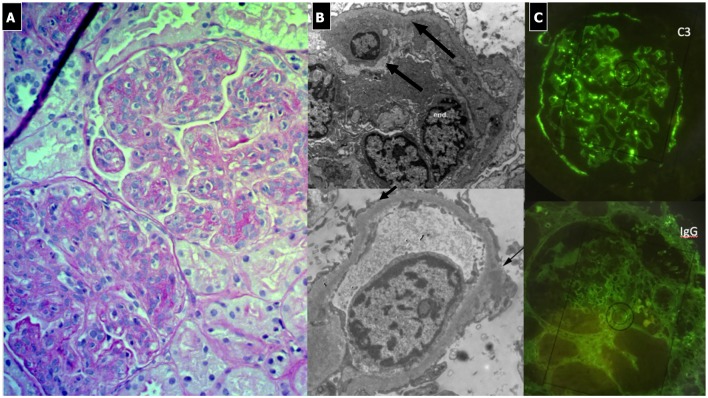
C3 Glomerulonephritis. **(A)** LM (PAS stain) shows lobular pattern with evidence of diffuse endothelium-mesangial proliferative GN. **(B)** EM demonstrates the splitting of GMB due to mesangial interposition (arrows). The lower image shows the presence of low intensity electron-dense sub-epithelial deposits (arrow). **(C)** IF shows the presence of C3 dominant staining.

**Figure 6:**
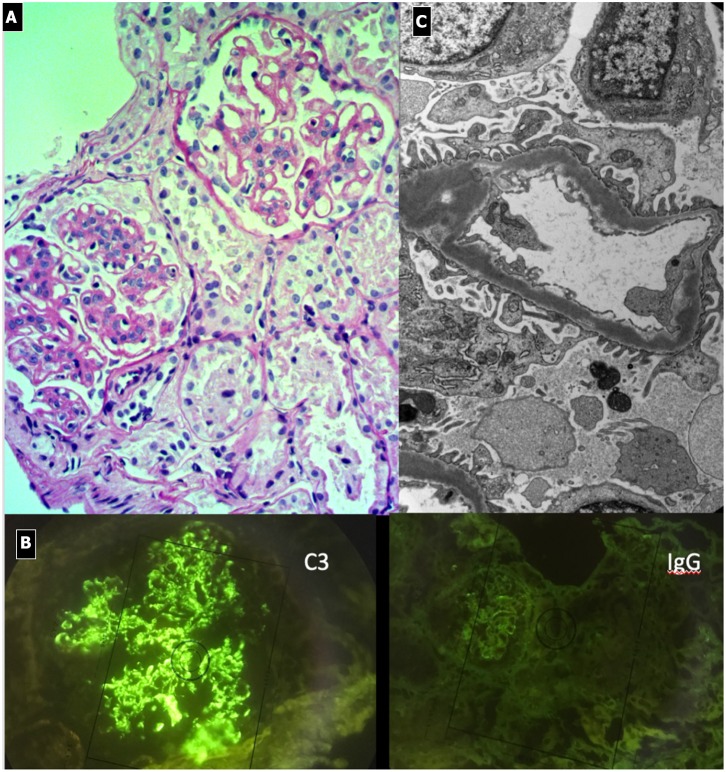
Dense Deposit Disease. **(A)** LM (PAS stain): global thickening of GBM associated with lobular pattern. **(B)** IF shows isolated C3 positivity (mesangial/capillary wall distribution). **(C)** EM, typical electron-dense deposits along the entire course of the glomerular basement membrane (ribbon-like appearance).

C3G show various histological patterns of glomerular injury (Figures 5, 6), including mesangial proliferative, diffuse endocapillary proliferative and crescentic glomerulonephritis (10, 101–103). The IF aspect represents the key diagnostic feature showing the presence of C3 dominant staining (C3 intensity at least twice that of any other immune reactant). In DDD, EM typically reveals the presence of electron-dense deposits along the entire course of the GBM (ribbon-like appearance) and occasionally along the tubular basement membrane and Bowman's capsule. Otherwise, C3GN EM is characterized by less intense and less well-defined electron-dense deposits (mesangial, sub-endothelial, intramembranous or sub-epithelial) (61). Humps, previously considered pathognomonic of PIGN, have recently been described in IC-MPGN and C3G on EM (104). It is interesting to note that PIGN can exhibit isolated C3 deposition on IF, particularly during the post-acute phase (14), when C3 amplification may be persistent and deposition of IgG decreases to undetectable levels (105). All these features indicate that PIGN, C3G, and MPGN could represent a spectrum of GN, with either primary or secondary complement activation, often triggered by infections (106). In this regard, glomerular staining of C4d (and rarely C1q), a by-product of CCP activity, is considered a useful tool in the identification of an IC-mediated mechanism, as observed in PIGN and IC-MPGN (106, 107).

#### Treatment

No adequate clinical trials evaluating the treatment of C3G are available. Recommendations and therapeutic algorithms are based on expert opinion and scanty published data (Table 3). Steroid monotherapy proved ineffective in adult patients with DDD in the only randomized clinical trial in the literature (18). The use of MMF was reported to be useful in C3GN (22), while the use of calcineurin inhibitors (31, 32) or rituximab (33, 34) were inefficacious. The identification of the role played by ACP activation has opened up new perspectives for the treatment of C3G. It is important to underline that the utility of biomarkers such as C5b-9 plasma levels in predicting treatment response and customizing therapy remains unclear (25). Eculizumab, a recombinant humanized monoclonal antibody directed against the C5 fraction of complement, was tested in DDD and C3GN pediatric patients (24) and in adult patients with crescentic/rapidly progressive GN (RPGN) (108) with good results. In any case, the optimal duration of eculizumab therapy in responders, and useful parameters in patient selection, remain unclear, though a limited treatment duration may be sufficient. The effective removal of C3NeF with plasma exchange proved ineffective (26), while partial or complete remission was reported in 17 out of 21 (81%) cases (27, 28). Case reports involving fresh plasma infusion claimed prevention of disease progression in 3 patients (2 DDD and 1 IC-MPGN) (27, 29, 30). Recently, more targeted agents have been proposed: C3 inhibitors (compstatin and staphylococcal C inhibitor), C5a inhibitors and C5aR antagonists (PMX-58 and AcePepA, soluble CD59, and clusterin) (35–39).

In conclusion, from a practical point of view, two main therapeutic lines have been suggested:

- KDIGO recommends the use of steroids and MMF for patients with moderate disease and intravenous methylprednisolone for severe disease, while insufficient data are available to recommend the use of eculizumab for rapidly progressive GN with isolated C3 deposits (21).
- Riedlet et al. (20) suggest a “wait-and-see” approach for patients with normal renal function without nephrotic range proteinuria. In the other cases, prompt treatment with fresh frozen plasma, plasma exchange or eculizumab, to restore complement function, is suggested (27, 30) adding steroids and MMF or rituximab in patients with positive C3Nef antibodies (23).

The use of RAAS blockers (single or double blockade) has been strongly suggested for reducing proteinuria (40, 83), despite the persistence of low C3 levels, because an increase in renal survival has been observed.

In our experience, an intensive course of immunosuppression is administered to patients with a urinary protein to creatinine ratio (uPr/uCr) >0.5–1 mg/mg in order to reduce renal inflammation and systemic autoimmune activation, which affects the complementary cascade. We thus prescribe 1 to 3 cycles of three boluses of methylprednisolone (15 mg/kg/dose), followed by oral prednisone (1 mg/kg every other day), eventually adding an immunosuppressive agent (MMF). We usually prescribe RAAS blockers after 3 months to properly assess the effectiveness of the induction therapy.

## Normocomplementemic Glomerulonephritis

### Glomerulonephritis Due to Abnormally Glycosylated IgA Deposition

Immunoglobulin A nephropathy (IgAN) and Henoch-Schönlein purpura nephritis (HSPN), also known as immunoglobulin vasculitis nephritis or anaphylactoid purpura nephropathy, are two pediatric glomerulopathies resulting from the glomerular deposition of an abnormally glycosylated IgA1, with mesangial proliferation (42).

### IgA Nephropathy

Immunoglobulin A nephropathy or Berger's disease, first described in 1968 by Berger and Hinglais, is the most common type of primary GN throughout the world in adolescents and young adults, with a male predominance (109, 110). It has a higher incidence in Asia and Australia (24–30% of all GN) and Europe (18%) than in the United States (2–10%): these differences are in part related to a different “biopsy policy” (110), as regards microscopic hematuria.

#### Clinical and Laboratory Features

Clinical manifestations vary from asymptomatic microscopic hematuria to RPGN. Typically, the recurrence of gross haematuria coinciding with a febrile infection is the presenting symptom. Urinary abnormalities can be persistent between acute events or may represent the only clinical finding. A renal biopsy is necessary to determine the diagnosis (111).

#### Natural History and Prognosis

Although initially considered a benign disease, 10% of children and up to 40% of adults with IgAN progress to end stage kidney disease within 20 years (42, 50), with a risk of recurrence after kidney transplant of up to 50% (112). Several clinical and histological risk factors for progressive kidney damage have been identified, both in adults and children, hypertension at renal biopsy and proteinuria being the most relevant (53, 113–117). In the Validation of the Oxford Classification of IgAN (VALIGA) study (>1,000 patients from 13 European countries), the threshold of persistent proteinuria as a risk factor for a 50% reduction in GFR or need for dialysis was >0.5 g/day (118). Conversely, proteinuria at onset was not associated with bad prognosis, probably because it represents the consequence of the acute inflammation, reversible with steroid therapy. Gross hematuria, per se, is not prognostic (119), but persistence of severe microscopic hematuria has recently been considered a risk factor (120). Glomerular filtration rate at biopsy is a predictor of the need for dialysis in adults (118, 121–124). Adverse histology includes the presence of extensive (>25%) fibrosis, crescents and mesangial proliferation. The predictive value of mesangial hypercellularity and crescents is attenuated by immunosuppression, indicating that proliferative lesions are steroid-responsive, particularly in children (112, 125). Finally, the presence of C4d on renal biopsy has been recently proposed as a new negative prognostic biomarker (126, 127).

#### Pathogenesis

IgAN has recently been redefined as an immune complex-mediated disease with a multi-hit pathogenesis, in which genetic and epigenetic factors play a role in association with the intestinal microbiota (gut-kidney axis) and the mucosal-associated lymphoid tissue (50, 109–111).

Genetic susceptibility plays a major role in this pathway and it has to be considered as “the first hit.” Mutations in genes encoding glycoprotein-N-acetylgalactosamine-3-β-galactosyltransferase (C1GalT1), an enzyme involved in the post-translational galactosylation of circulating lymphocytes (49, 109), B-cell Activating Factor (BAFF) and A PRoliferation-Inducing Ligand (APRIL) play a major role (109). On the other hand, mutations in CFHR1–3 genes appear to have a protective role, because the absence of these complement regulatory proteins (CFHR1–3) increases CFH activity with a more effective inhibition of ACP and a reduced production of C3a (111).

Following antigen exposure (environmental or dietary antigens, mucosal infections), the innate immune system is excessively activated by the Toll-like receptors, B-cell activating factor and proliferation-inducing ligand signaling (49). As a result of mis-homing of a proportion of mucosal B cells to systemic sites, poorly O-galactosylated-IgA1 (Gd-IgA1) is secreted into the circulation by gut lymphocytes with autoantibody production against the Gd-IgA1 hinge region and nephritogenic immune complex formation (49, 50, 109–111). The glomerular deposition of these ICs is favored by the dysregulation of CD71, a receptor on mesangial cells stimulated by soluble CD89, which is involved in IgA complex amplification, the activation of mesangial cells and pro-inflammatory cytokine (IL-6, IL-8, IL-1β, TGF-β) release. This cascade of events alters GBM permeability with the consequent damage of podocytes and proximal tubule epithelial cells. Finally, the activation of the ACP and the lectin complement pathway (LCP) completes the process of renal damage, contributing to glomerulosclerosis and tubulointerstitial fibrosis (49, 50), and playing a crucial role in leukocyte infiltration of the kidney and an essential role in disease progression (109).

#### Renal Histopathology

**Figure 7:**
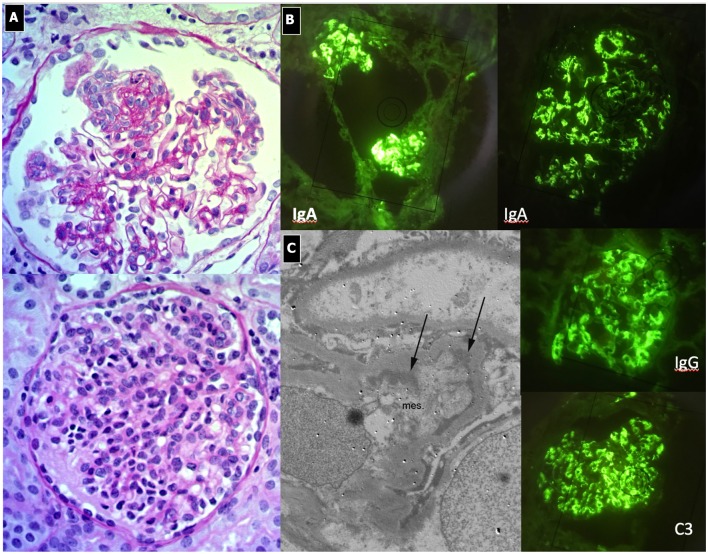
IgA Nephropathy. **(A)** LM (PAS stain): Two glomeruli show diffuse and global mesangial proliferative lesions, with endocapillary proliferation. **(B)** IF, global and diffuse mesangial IgA deposits, associated with C3 and IgG deposition. **(C)** EM, presence of paramesangial immune deposits (arrows).

Immunofluorescence findings of mesangial IgA deposits, commonly associated with C3 deposition, is the key diagnostic feature (Figure 7). On LM, findings vary from minimal mesangial expansion to diffuse proliferative lesions with crescents and/or widespread sclerosis. More often, there is an increase in the mesangial area, due to the expansion of mesangial cells, matrix, and deposits. Endocapillary proliferation may be present (focal segmental or diffuse) and is typically associated with the extension of deposits into the subendothelial areas of the peripheral loops. In severe cases, segmental necrosis and crescents may be seen. Chronic cases are characterized by segmental sclerosis with proportional tubular atrophy and interstitial fibrosis, the presence of which is confirmed by EM.

The recently updated 2016 Oxford classification (MEST-C) (125) considers each pathological feature separately. The VALIGA study demonstrated the presence of mesangial hypercellularity (M), endocapillary hypercellularity (E), segmental glomerulosclerosis (S), tubular atrophy/interstitial fibrosis (T), and glomerular crescent formation (C) (49, 112) as independent predictors for developing progressive renal disease in adults (112, 118, 125).^.^ In a cohort of 174 patients under 18 years of age, the presence of mesangial proliferation (M1) was the sole predictor of poor outcome with a survival rate of 46% at 15 years vs. 100% with no proliferation (M0) (128).

#### Treatment

In the past, IgAN was considered by many nephrologists to be a benign condition not requiring treatment. For this reason, no evidence-based treatment guideline for pediatric IgAN is available. Although many therapeutic trials have been conducted, in 2006, Appel concluded that there was no consensus on the best treatment (129, 130).

Over the last 10–15 years, several randomized controlled studies in adult patients have been published, contributing to the 2012 KDIGO guidelines (Table 3).

IgAN with minimal glomerular abnormalities can spontaneously remit. When proteinuria is < 0.5 g/24h, with normal eGFR, RAAS blockers are the recommended therapy (41). The most effective treatment for patients with normal, or almost normal, renal function and moderate proteinuria should be a combination of RAAS blockers and corticosteroids (43–46). It is important to underline that the KDIGO guidelines discourage corticosteroid use in patients with an eGFR <30 ml/ min (131), given the presence of chronic lesions on histology.

Cyclophosphamide is reserved for rapidly progressive IgAN, reducing the frequency of end-stage renal failure (47). In randomized prospective studies, MMF was ineffective in the short-term, but provided renoprotection in the long-term in adults (52).

In pediatric patients with IgAN, we prescribe an observational follow up (every 6 months) for those with microscopic hematuria or recurrence of gross hematuria without persistent proteinuria. Children with mild proteinuria (uPr/uCr <0.5–1 mg/mg) are treated with RAAS blockers and/or ARBs, with dosage titration. In patients with uPr/uCr >0.5–1 mg/mg, 3 daily pulses of methylprednisolone at months 1, 3, 5, plus oral prednisone 0.5 mg/kg every other day for 6 months, followed by tapering for 6 months with the addition of RAAS blockers is recommended. We recommend cyclophosphamide in patients with the rapidly progressive form.

Rituximab is not effective in IgAN patients, despite systemic CD20 B cell depletion. Temporary benefit in stabilizing renal function or proteinuria was observed in two patients with rapidly progressive IgAN treated with Eculizumab (49). Tonsillectomy, polyunsaturated fatty acids and statins have been suggested but are not widely accepted forms of treatment (50, 51). New immunomodulatory strategies have been attempted with good preliminary results (49).

### Henoch–Schönlein Purpura Nephritis (HSPN)

Henoch–Schönlein purpura nephritis (HSPN) is a GN secondary to a systemic disease and is characterized by leukocyte infiltration of the small blood vessel walls, with added IgA1 immune complex deposition (42). Henoch–Schönlein purpura is the most common pediatric vasculitis in Western countries (estimated incidence rate of 30/100,000 children per year) that usually affects children from 3 to 10 years of age, during the autumn and winter months. A higher incidence has been reported in white, Asian and Hispanic populations than in black populations (132). Male gender, age >10 years, severe gastrointestinal symptoms (abdominal pain, gastrointestinal bleeding, severe bowel angina), arthritis/arthralgia, persistent purpura or relapse, white blood cells >15 x 109/L, platelets >500 x 109/L, elevated antistreptolysin O and low C3 concentrations are described as risk factors associated with renal involvement in HSP (133).

#### Clinical and Laboratory Features

Henoch–Schönlein purpura is a self-resolving necrotizing vasculitis, triggered by upper respiratory tract infections. It is clinically characterized by a non-thrombocytopenic palpable purpuric rash on the ankles and lower legs associated with articular (arthralgia/arthritis, edema), gastrointestinal (bloody stools, abdominal pain) and renal disease (gross or microscopic hematuria) (42). The European League Against Rheumatism (EULAR), the Pediatric Rheumatology International Trials Organization (PRINTO) and the Pediatric Rheumatology European Society (PRES) (EULAR/PRINTO/PRES) defined the diagnostic criteria for HSP diagnosis (Table 4) (134).

**Table 4:** EULAR/PRINTO/PRES diagnostic criteria for Henoch-Schönlein Purpura diagnosis.

**Criterion**		**Frequency (%)**
Palpable purpura plus one of the following	∎Symmetric purpura, mainly over extremities following gravity ∎ Exclude thrombocytopenia	100
Abdominal pain	∎Colic-like, postprandial ∎ Nausea, gastrointestinal bleeding ∎ Intussusception, infarction, perforation	50
Histopathology	∎Immune complex vasculitis ∎ Proliferative glomerulonephritis (IgA deposition)	-
Kidney involvement	∎Proteinuria >0.3 g/24 h or ∎ Albumin/creatinine ratio >30 mmol/mg ∎ Micro-hematuria ∎ Arterial hypertension ∎ Nephritic or nephrotic syndrome	20–40
Joint involvement	∎Arthritis, mostly ankles and/or knees	70

Renal manifestations occur in up to 80% of pediatric patients, typically within 6 months of disease onset, ranging from urinary abnormalities (microscopic or gross hematuria with or without proteinuria, in 50% of cases) to nephritic/nephrotic syndrome (20% of cases), usually associated with acute renal failure and arterial hypertension (135). For all these reasons, urine testing to monitor kidney involvement is mandatory (48). We suggest performing a urinary dipstick weekly for 1 month, every 2 weeks for 2 months and monthly for 3–6 months. A renal biopsy is suggested in patients with persistent proteinuria (>1 mg/mg for 4 weeks) or impaired GFR at onset (110, 135).

#### Natural History and Prognosis

Renal prognosis is good in most cases as the condition eventually resolves. Children with persistent renal inflammation and crescentic glomerulonephritis (>50%) may progress to ESRD in up to 20% of cases (48). Glomerular crescents, tubulointerstitial or chronic lesions are the prognostic factors most correlated with poor outcome (135).

#### Renal Histopathology

The pathologic lesions are similar to those seen in other IC–mediated diseases, in particular lupus nephritis. The findings range from pure mesangial proliferative GN to focal segmental necrotizing GN. Sometimes a pattern of MPGN can be present, often associated with crescents. The glomerular inflammatory lesions have been conventionally classified by the International Study of Kidney Disease in Children (ISKDC) (Table 5) (136). The Oxford classification has also been proposed, but has not yet been validated (135). Immunofluoresence demonstrates mesangial to peripheral capillary deposition of IgA, which is a clue to diagnosis.

**Table 5:** Classification of renal lesions in course of Henoch-Schönlein Purpura by the International Study of Kidney Disease in Children (ISKDC).

**ISKDC grade**	**Description**
Grade I	Minimal alterations
Grade II	Mesangial proliferation
Grade III	Proliferation or sclerosis with <50% crescents ((a) focal or (b) diffuse)
Grade IV	Mesangial proliferation or sclerosis with 50–75%, crescents ((a) focal or (b) diffuse)
Grade V	Mesangial proliferation or sclerosis with > 75% crescents ((a) focal or (b) diffuse)
Grade VI	Membranoproliferative like glomerulonephritis

#### Pathogenesis

Similarly to IgAN, it has been hypothesized that HSPN has a multi-hit pathogenesis, triggered by an abnormal immune response to a number of antigenic stimuli (bacteria such as *H. pylori, S. pneumoniae, H. influenza* or viral agents) in genetically susceptible subjects (137). Galactose deficient-IgA1 is the main component of large immune deposits, together with IgG autoantibodies and CD89, which are deposited in the mesangium leading to inflammation of the glomerulus, as previously described in IgAN. Genetic association studies identified HLA-DRB1^*^07 as a protective locus and HLA-DRB1^*^01 and HLA-DRB1^*^11 as predisposing loci (132). Other gene polymorphisms involved in cytokine and chemokine production, the renin-angiotensin system, aberrant IgA1 glycosylation, neoangiogenesis, nitrite oxide production, complement activation, and endothelium activity regulation have all been implicated in HSPN susceptibility and severity (137, 138).

#### Treatment

Since 2012, the KDIGO guidelines have proposed the same treatment for IgAN and HSPN, based on disease severity with the optimal proteinuria target <0.5 g/24 h/1.73 m (2). However, HSPN has more acute histological features, while IgAN has a more chronic imprinting, Therefore, the use of the same therapy scheme is not universally accepted (42). It must be remembered that KDIGO recommendations are based on patients with IgAN.

For this reason, in 2019, the European Consensus of pediatric rheumatologists produced new recommendations for HSP (Table 3). They recommend corticosteroids and immunosuppressive agents as first-line therapy for mild/moderate and severe HSPN in the acute phase in order to prevent CKD, in association with RAAS blockers when proteinuria is present. When GFR is normal and uPr/uCr <2,5mg/mg, oral prednisolone is indicated, plus steroid sparing agents (azathioprine, MMF, cyclosporine) when there is a lack of response to oral steroids. Intravenous cyclophosphamide with corticosteroids is recommended in children with severe HSPN (>50% crescents on biopsy and impaired GFR or severe persistent proteinuria) followed by azathioprine and MMF. The use of rituximab is not recommended (48).

We employ 3-daily pulses of methylprednisolone (15 mg/kg/dose, months 1,3,5) followed by oral prednisone (0.5–1 mg/kg every other day) for 6 months, and then tapering, as the first-line treatment. We consider immunosuppressive drugs (cyclophosphamide in the induction phase and MMF/azathioprine in the maintenance phase) for severe/crescentic or steroid-resistant cases. We use RAAS blockers after 3 months, in order to evaluate the efficacy of the induction therapy.

### Glomerulonephritis Due to *in situ* Immune Deposits: Membranous Nephropathy

Membranous nephropathy (MN) is a non-inflammatory organ-specific autoimmune disease affecting the glomerulus, resulting in the formation of immune deposits on the outer aspects of the GBM. Membranous nephropathy can occur in all age groups, from infancy to old age. In the adult population, it is the commonest cause of NS, while the percentage drops to 3% in children under 13 years of age and rises to 18% in adolescents (139). Data from the ISKDC demonstrates an incidence of 1.5% in children with NS (140).

In the majority of children, it is secondary to infections, particularly hepatitis B in endemic areas, and systemic diseases, mainly SLE. In the remaining cases, it is defined as “idiopathic.” Malignancy associated MN is very rare in the pediatric population.

#### Clinical and Laboratory Features

The most frequent (40–75%) clinical pattern at onset is NS (139). Proteinuria is present in all cases of MN, even if non-nephrotic. Kidney function and blood pressure are generally normal. The presence of hematuria (70% microscopic, 30% gross) is more common in children than in adults (139, 141).

#### Natural History and Prognosis

Evolution is extremely variable: spontaneous remissions occur in 35–50% during the first 2 years (142). Progression to ESRD, although slow and less frequent than in the adult population, is seen in 20% of children with persistent NS. In 2008, the North American National Report on Pediatric Kidney Disease indicated that MN is responsible for 0.4% of pediatric ESRD and 0.5% of pediatric causes of CKD (143). No prognostic factors have been identified in pediatric patients. Adult studies showed that anti-PLA2R titers correlate with disease activity and patient outcomes: (58, 144, 145) a low titer predicts spontaneous remission, while a high titer (>275 IU/ml) is associated with NS, a high risk of relapse and progressive loss of renal function. Preliminary data suggest that the same holds true for anti-THSD7A levels (146). Likewise, the nature of antibodies influences disease severity: those targeting the CTLD1 and CTLD7 domains of PLA2R1 are detectable in older patients with more severe proteinuria, rare spontaneous remission and higher risk of progression to ESRD (147). Other negative prognostic factors that have been reported at disease onset include low molecular weight urinary proteins, elevated serum creatinine, severity of proteinuria and presence of tubular-interstitial fibrosis at renal biopsy (148).

#### Pathogenesis

Our understanding of the pathogenetic mechanisms goes back to the experimental model of Heymann nephritis and to investigations that demonstrated glomerular antigens in the extra-membranous area. In humans, the first target antigen to be discovered was neutral endopeptidase (NEP), a protein expressed on podocytes. During pregnancy, women with NEP deficiency develop anti-NEP antibodies that cross the placenta and bind to the NEP expressed on fetal podocytes. After birth, a congenital form of NS develops (149–153). Recently, the phospholipase 2 receptor (PLA2R) glycoprotein expressed by podocytes has been identified as the most frequent target of autoantibodies (anti-PLA2R) in up to 70–80% of adult cases of MN, but in only 40–50% of children, thus leaving the door open for other causal antigens. Another human podocyte antigen, the thrombospondin type 1 domain-containing 7A protein (THSD7A), has been more recently identified in 5–10% of patients (146) and is associated with malignancies (154). In children <5 years of age, the presence of circulating anti-bovine serum albumin antibodies (anti-BSA) and the finding of bovine serum albumin (BSA) and IgG anti-BSA in subepithelial deposits implies the pathogenetic role of an exogenous glomerular antigen (155). It has been postulated that the metabolic action of the intestinal microbiota leads to cationic modifications of the BSA, which can then cross the GBM and accumulate on its subepithelial anionic side (156).

#### Renal Histopathology

**Figure 8:**
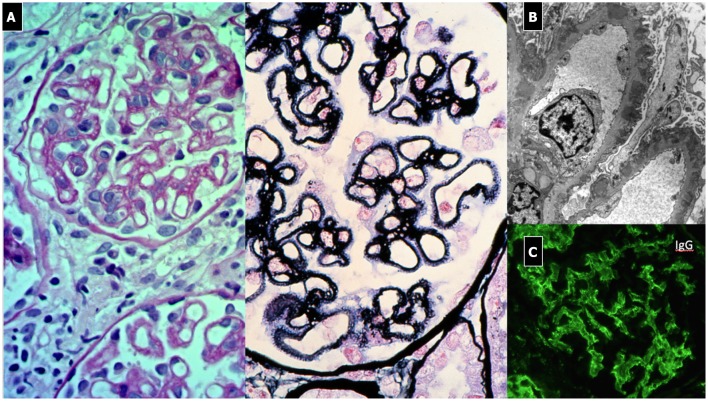
Membranous Nephropathy. **(A)** LM shows (PAS stain) regular and diffuse thickening of GBM without endothelium-mesangial proliferation and (silver stain) the GBM matrix reaction due to the presence of deposits (spikes). **(B)** EM shows sub-epithelial deposits with spikes of GBM that begin to encircle the deposits (Stage 1). **(C)** IF: diffuse and granular subepithelial deposits of IgG along the capillary wall (garland pattern).

The typical histopathological lesion is thickening of the GBM, related to the presence of immune deposits (Figure 8). During the early phases, a more rigid-appearing capillary wall may be present, with no surrounding GBM reaction (spikes). Initial immunoglobulin deposition appears like “holes” in the silver staining, as they do not stain with silver (Stage 1). As deposits persist, the GBM matrix reaction produces small spike-like protrusions (Stage 2) visualized by silver staining, followed by the matrix encircling the deposits resulting in a lace-like splitting of the GBM (Stage 3). Finally, the deposits are reabsorbed (Stage 4). Additional lesions include segmental sclerosis, interstitial fibrosis, and tubular atrophy, generally associated with a worse prognosis. Crescents are very rare and should raise the suspicion of SLE or anti-GBM nephritis. Also, the presence of mesangial deposits suggests a secondary form (Class V Lupus Nephritis). Diffuse and granular subepithelial deposits of IgG and, less frequently, C3 along the capillary wall are present on IF. Mesangial deposits are typically present in secondary forms. On EM, deposits corresponding to the stage of the disease are visualized, with a variable surrounding GBM reaction (139).

#### Treatment

Spontaneous remission occurs in up to a third of patients, even if the initial presentation includes massive proteinuria (157). This finding supports the 6-month “wait and see” policy, unless a rapid loss of GFR is present or life-threatening NS complications occur (53). Thereafter, patients with persistent proteinuria should be treated with supportive anti-proteinuric therapy (RAAS blockers), although less effective than in other chronic nephropathies (55, 56). Immunosuppressive drugs are utilized in patients with a severe disease or steroid-resistant nephrotic proteinuria (Table 3). Cyclophosphamide and chlorambucil, and, less frequently, cyclosporine, for at least 6 months all produce positive results albeit with greater side effects (57). Recently, the efficacy of rituximab and other anti-CD20 monoclonal antibodies has been reported in adults and children, with fewer side effects (58, 59, 158). However, rituximab therapy may fail in 25–30% of patients with NS because of an escape phenomenon vs. anti-CD20 treatment (57).

In our patients older than 5 years of age with anti-PLA2R autoantibodies, we start treatment with rituximab (2 doses of 375 mg/sm administered 14 days apart) and monitor levels of antibodies and CD20-cells. In children younger than 5 years and in older children without autoantibodies we prefer steroids (prednisone 2 mg/kg for 4 weeks than slowly tapered in 3–6 months) and cyclosporin (3–5 mg/kilogram of body weight for 12 months).

### Anti-neutrophil Cytoplasmic Antibody-Associated Vasculitis

Anti-neutrophil cytoplasmic antibody (ANCA)-associated vasculitis (AAV) is characterized by the destruction of small and medium-sized arterial vessels with few or no immune deposits, in the presence of circulating autoantibodies toward the cytoplasmic region of the neutrophil (ANCA) predominantly against proteinase-3 (PR3) and myeloperoxidase (MPO). Very rare cases are negative for ANCA, but they are considered in the same spectrum of the disease. Anti-neutrophil cytoplasmic antibody-associated vasculitis includes 3 different entities: granulomatosis with polyangiitis (GPA) or Wegener's granulomatosis; eosinophilic granulomatosis with polyangiitis (EGPA) or Churg-Strauss syndrome; microscopic polyangiitis (MPA). The emerging importance of ANCA serology in predicting outcome and risk of relapse has led to some new terminology. The new nomenclature considers the autoantigen involved (MPO/PR3) adding the relevant clinical manifestations, preferably using the terms GPA/MPA/EGPA, where present (e.g., PR3-AAV with clinical manifestations of GPA) (159).

The overall incidence is of 0.5–2 per million children per year in Europe, making AAV a rare disease (160, 161). A female predominance and onset in early adolescence (11–14 years) have been described (162).

#### Clinical and Laboratory Features

In 2008, the EULAR/PRINTO/PReS societies validated the classification of GPA (Table 6) (25, 160), with common clinical manifestations, even if single organ involvement has been reported. No specific criteria for childhood-onset MPA (Table 7) or EGPA have been reported (25, 160), Non-specific findings such as fever, fatigue, anorexia, and weight loss may precede the systemic manifestations. Other symptoms may include gastrointestinal, mucosal and skin lesions, ocular abnormalities, arthralgia, nervous system involvement, and deep venous thrombosis (163).

**Table 6:** According to EULAR/PRINTO/PReS criteria, the definition of GPA requires three out of six criteria (Sensitivity 93%, Specificity 99%).

**Criterion**	**Pattern**
Renal involvement	Proteinuria, hematuria, or red blood cell casts
Positive histopathology	Granulomatous inflammation within the wall of an artery or perivascular or extravascular area
Upper airway involvement	Nasal discharge or septum perforation, sinus inflammation
Laryngotracheobronchial involvement	Subglottic tracheal or bronchial stenosis
Pulmonary involvement	Chest X-ray or CT-scan evidence
ANCA positivity	By immunofluorescence or by Enzyme-Linked Immuno-Sorbent Assay (ELISA) PR3-ANCA or MPO-ANCA

**Table 7:** Classification criteria of MPA according to the Annals of the Rheumatic Diseases of the BMJ.

**Criterion**	**Score**
Pauci-immune glomerulonephritis	+3
Blood nasal discharge, ulcer, crusting, congestion or blockage, septal defect/perforation	−3
pANCA or MPO-antibody positivity	+6
Fibrosis or Interstitial Lung Disease on chest imaging	+3
cANCA or PR3-antibody positivity	−1
Eosinophil count ≥1 ×10 (9)/L	−4

*Total score ≥6 is needed (Sensitivity 87%, Specificity 96%)*.

The most common clinical presentation of GPA involves the upper respiratory tract (up to 92% of adult patients). This feature might be shared with EGPA, but is rarely seen with MPA. Involvement of the upper respiratory tract (rhinitis, sinusitis, nasal crust, epistaxis, otitis media, sensorineural or conductive hearing loss) precludes the diagnosis of MPA (164). Subglottic tracheal stenosis is a severe complication and that occurs 5 times more frequently in pediatric patients than in adults (163).

Lower respiratory tract involvement is a shared feature in all AAV subtypes (cough, wheezing, hemoptysis, and bronchial stenosis). Catastrophic pulmonary hemorrhage may also occur in up to 42% patients in MPA (164). Eosinophilic granulomatosis with polyangiitis predominantly affects the airways, less frequently the skin, heart, gastrointestinal tract, or nervous system. Renal involvement is the most important concern. It is a severe manifestation in GPA and MPA, often leading to ESRD and thus causing significant morbidity and mortality. It commonly manifests as a pauci-immune necrotizing GN, with a variety of clinical phenotypes, ranging from rare cases of “slowly progressive” forms of GN, characterized by isolated urinary abnormalities (microscopic hematuria, blood-red casts and/or proteinuria), to a more frequent manifestation including acute severe kidney injury often requiring renal replacement therapy in the context of a crescentic glomerulonephritis (a rapidly progressive form of AAV GN) (162). Usually, AAV leads to a normocomplementemic form of GN.

Renal involvement in AAV is common. In MPA, it is present in 94–100% of patients at onset (164), whereas GPA renal involvement has been noted in 50–100% of children at onset (163). To date, no pediatric case report has described renal injury in EGPA (25, 160).

#### Natural History and Prognosis

Prompt diagnosis is closely correlated with a favorable prognosis. If organ-specific findings are only mild, a delay in diagnosis may result in chronic, irreversible lesions. Renal disease is the most relevant determinant of long-term prognosis and morbidity in children: more frequent and severe in MPA than GPA, mild and not progressive in EGPA (ESRD up to 40% in MPA vs. 10% in GPA) (60). ANCA serology (MPO or PR3) is a relevant prognostic factor as regards outcome and relapse risk (18). However, serum C3 consumption and/or C3 and immunoglobulin glomerular focal deposition and sclerotic lesions predict an adverse outcome (60, 81). Patients with MPA and pulmonary-renal involvement and high MPO-ANCA titers progress to ESRD within 1 to 5 years (81). If untreated, GPA has a mortality rate of 100% in the first year, while it recurs in up to 60% of treated patients (17). The mortality rate in treated children is <5–10% (60). Kidney transplantation is recommended during periods of clinical and serological remission, and the disease can recur in the graft.

#### Pathogenesis

A multifactorial pathogenesis is involved in the development of AAV: genetic and epigenetic factors interact with environmental (infections, drugs and air pollutants), hormonal and immunological alterations. Some HLA-DP alleles are associated with a major risk for developing GPA. Mutations in the SERPINA1 gene encoding for alpha1-antitrypsin, the principal inhibitor of PR3, and in PR3 encoding gene (PRTN3) may predispose to GPA. Conversely, MPA is associated with HLA-DQ polymorphisms (160, 165).

High levels of MPO and PR3 are noted on the surface of primed circulating neutrophils, where they are most exposed to the action of autoantibodies (164). Regardless of the trigger, a series of events occurs leading to B cell dysregulation which, in association with an imbalance between helper and effector T cells, leads to ANCA production and neutrophil activation with consequent tissue damage and vessel inflammation. A role of CAP has been demonstrated (60) in neutrophil activation and renal damage (166).

#### Clinical Meaning of Serum ANCA

Serology, namely ANCA analysis, is needed to differentiate GPA from MPA, with a high sensitivity (93%) and specificity (90%). Granulomatosis with polyangiitis usually shows anti-PR3 ANCA positivity in 60–80% of cases, with MPA anti-MPO ANCA in 80–90% and EGPA in 35–40% of cases, respectively (160, 162). In limited disease forms, or in EGPA, ANCA screening is often negative (160). Isolated renal vasculitis has been described with 80% MPO-ANCA positivity, causing a rapidly progressive GN (162).

The pathogenetic role of ANCA has been questioned. Murine *in vivo* studies demonstrate that it is possible to induce GN by transferring anti-MPO antibodies. Furthermore, the association between PR3-AAV and both polymorphisms in HLA and a mutation in PR3 encoding gene suggest a pathogenetic role. Finally, the relationship between ANCA titer and disease activity is not clear-cut, the evidence that some antibody-depletion therapies, such as plasma-exchange and anti-CD20 monoclonal antibody are efficacious in AAV support the pathogenetic hypothesis. It is worth underlining that some patients without AAV may have significant ANCA titers. The antibodies appear directed against “non-pathogenetic” epitopes (166).

#### Renal Histopathology

**Figure 9:**
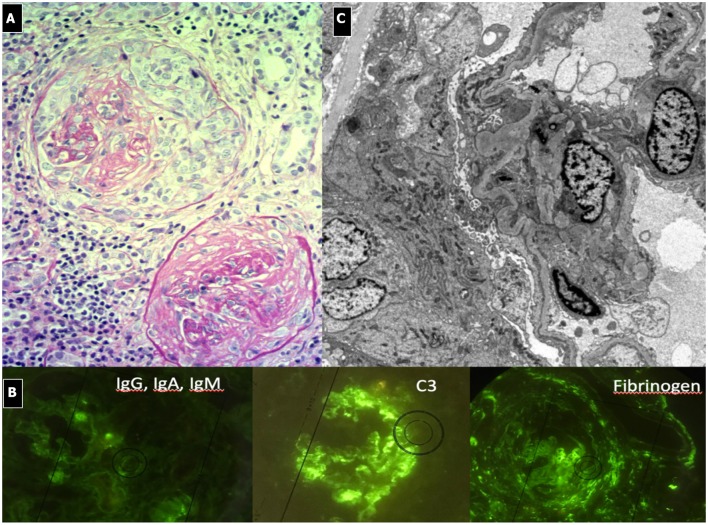
ANCA Associated Vasculitis Nephritis. **(A)** Diffuse crescentic, sclerotic and focal necrotizing GN. Diffuse interstitial flogistic infiltration. One glomerulus is almost completely fibrotic. **(B)** pauci-immune IF pattern with evidence of fibrinogen deposition due to diffuse hyalinosis. C3 focal deposition. **(C)** EM, diffuse sclerosis.

The histological picture typically shows a pauci-immune, focal necrotizing and crescentic GN (162, 165). Immunofluoresence is typically negative (Figure 9). Recently, a histopathologic classification has been validated for the pediatric forms, which correlates with renal function at 2 years: focal lesions demonstrated better outcome compared to crescentic/mixed lesions and sclerosis (162). Tubularsclerotic lesions, C3 and immunoglobulin focal deposition on IF are also markers of adverse outcome (160).

#### Treatment

The EULAR-ERA EDTA recommendations and the SHARE project indicate intravenous high dose steroids and cyclophosphamide for 3–6 months as the first-line therapy in children (Table 3). Rituximab is an alternative for children with refractory or relapsing disease (60, 61). Mycophenolate mofetil has been trial tested as an induction therapy with positive results in children with more frequent relapses. Plasma exchange is an optional treatment in rapid progressive renal failure or when alveolar hemorrhage is present. In the maintenance phase, a combination of low-dose steroids and azathioprine, rituximab or MMF can be used for an undefined follow-up period (160, 167). With adequate therapy, remission can be achieved in 70–100 % of patients (168). TNF-alfa receptor blocker (etanercept), TNF monoclonal antibody (infliximab), cytotoxic T lymphocyte-associated antigen 4 (CTLA-4) IgG Fc fusion protein (abatacept), human anti-CD52 monoclonal antibody (alemtuzumab), antiIL-6 receptor human monoclonal antibody (tocilizumab), and anti IL-5 human monoclonal antibody (mepolizumab) have been tested in adults with promising results, but they are recommended in children to date (169).

Our protocol involves an initial course of intravenous boluses of methylprednisolone (15–30 mg/kg/dose maximum 1 gr for three consecutive days), followed by oral prednisone (1 mg/kg/day) for 4 weeks, and then slow tapering. The steroids are accompanied by cyclophosphamide (orally 2–3 mg/kg/day for 8–12 weeks, with a maximum 150 mg/kg cumulative dosage, or intravenously 500 mg/dose every 2 weeks repeated 6 times) or by plasma exchange, depending on the severity of disease. We prefer to utilize MMF rather than azathioprine in the maintenance period, in association with progressively tapered oral steroids.

## Rapidly Progressive Glomerulonephritis

This definition comprises the spectrum of abovementioned clinical conditions and is characterized by a rapid onset of progressive acute renal failure, in combination with a nephritic syndrome. The pathognomonic histological picture is crescents in more than 30% of the glomeruli, overlapping the principal features of an active GN. Complement status depends on the type of RPGN: elevated levels are found in systemic vasculitis, otherwise levels are decreased in SLE and other forms of IC-related GN (170). It is important to consider that, generally speaking, RPGN has to be considered as a sub-class of the abovementioned form of GN. For this reason, the most appropriate classification of RPGN is based on the presence, localization, and extent of deposits on IF. Three main forms are recognized: 1. IC-mediated, 2. anti-GBM mediated and 3. pauci-immune forms, often associated with ANCA.

1*. IC-RPGN* is the most severe clinical and histological form, particularly in children with PIGN, IgAN, IgAV and lupus nephritis. Around 10–15% of PIGN present as a rapidly progressive form. In the treatment of the rapidly progressive forms of these IC-related RPGN, the use of cyclophosphamide must be considered (see paragraph on the specific forms of GN).

2. *Anti-GBM RPGN* (*Goodpasture syndrome)* has an incidence of approximately 0.5 to 1 per million per year in adults and is even more rare in children (171). Reported pediatric cases are very scarce (*n* = 31) and show a prevalence in girls (M/F sex ratio, 1:4) and mean age at diagnoses of 9.2 ± 4.6 years (172). Goodpasture syndrome (GS) typically has a bimodal distribution with a pediatric peak between 10 and 20 years of age and a second peak after 60 years of age. Clinical manifestations can start following a triggering event (upper respiratory infection, smoking, hydrocarbon exposure, or influenza). Symptoms can be nonspecific (malaise, weight loss, fever, and arthralgia) or include severe renal and pulmonary manifestations. Kidney disease may occur independently from pulmonary disease and can range from hematuria and proteinuria to rapidly progressive renal failure. Pulmonary involvement, if present, may precede renal symptoms by weeks to months. Hemoptysis associated with respiratory failure is very common, due to the typical pulmonary bleeding. In sub-clinical cases, anemia and iron deficiency can be evident (173).

**Figure 10:**
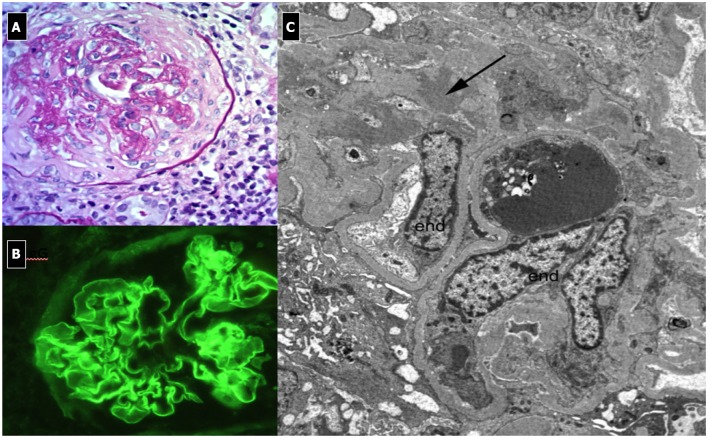
Rapidly Progressive Glomerulonephritis due to anti-GBM antibodies. **(A)** LM (PAS Stain); membranoproliferative, crescentic and sclerotic pattern associated with interstitial infiltration of leukocytes (endo-extracapillary pattern). **(B)** IF: Linear deposits of IgG along the capillary wall. **(C)** EM: shows no deposits and the presence of sclerosis.

The current mortality rate is about 30% in children, mainly due to severe pulmonary hemorrhage. Early treatment improves survival rates. Prognosis for renal recovery is worse in the presence of oliguria, presenting creatinine >5 mg/dl or renal biopsy showing >50% crescent formation within glomeruli at time of diagnosis or evidence of diffuse chronic damage (174). No residual pulmonary deficit or fibrosis is noted once the patient has recovered from the acute presentation. Though very rare, recurrence of the disease can occur, Goodpasture syndrome is associated with anti-GBM antibodies that localize in NC1 domain of the alpha3 chain domain of collagen IV, present in the GBM of kidneys and pulmonary alveoli. Linear deposits of IgG along the capillary wall are present on IF, while on LM, the glomeruli show breaks of the GBM due to fibrinoid necrosis and the consequent development of cellular crescents (Figure 10). In the very early stages of the disease, crescents may not be apparent, even in patients who present with severe, life-threatening lung disease. Other features include: Bowman's capsule ruptures, periglomerular fibrosis, fibrocellular/fibrous crescents, interstitial lymphoplasmacytic infiltrates, particularly around crescentic glomeruli, interstitial fibrosis, and tubular atrophy. A granulomatous or giant cell reaction may be present. No deposits are seen on EM, as the deposits have the same density as the GBM.

The initial treatment of choice is plasmapheresis to remove circulating antibodies. Immunoadsorbtion is an effective alternative therapy. Immunosuppressive therapy could include corticosteroids and cyclophosphamide or rituximab to reduce antibody production. Low dose prednisone, azathioprine, or mycophenolate may be used for maintenance immunosuppression once remission is established (175, 176). It is important to monitor levels of Anti-GBM antibodies weekly, until two negative results have been obtained, and then monthly for up to 6 months.

3*. Pauci*-*immune RPGN* is characterized by a complete absence of findings on IF. This group comprises a number of inflammatory diseases involving small vessels, as described in the paragraph about AAV. The co-presence of both ANCA and anti-GBM antibodies is relatively rare and requires aggressive and early treatment due to the extremely severe course. For this reason, we strongly suggest monitoring for anti-GBM antibody appearance in patients with AAV in order to start early, appropriate treatment to improve prognosis.

## Author Contributions

AM: co-designed the structure of the review and responsible for the sections on hypocomplementemic glomerulonephritis, membranous nephropathy, ANCA-associated vasculitis, and drew up the format of the final text. JS: in particular for glomerulonephritis due to abnormally glycosylated IgA deposition. MG: selected papers of literature to be considered, designed the structure of the review and supervised it. GM: concepted and designed the review, supervised the entire process and edited the final draft.

## Conflict of Interest

The authors declare that the research was conducted in the absence of any commercial or financial relationships that could be construed as a potential conflict of interest.
